# Interfacial fracture toughness of universal adhesive systems treated with an antioxidant

**DOI:** 10.4317/jced.54188

**Published:** 2018-06-01

**Authors:** Pouran Samimi, Reihaneh Nazem, Farinaz Shirban, Maryam Khoroushi

**Affiliations:** 1 DDS, MS. Associated Professor, Dental Materials Research Center, Department of Operative Dentistry, Isfahan University of Medical Sciences, Isfahan, Iran; 2 DDS, MS. Assistant Professor, Department of Operative Dentistry, Ghazvin University of Medical Sciences, Isfahan, Iran; 3 DDS, MS. Assistant Professor, Dental Research Center, Department of Orthodontics, School of Dentistry, Isfahan University of Medical Sciences, Isfahan, Iran; 4 DDS, MS. Professor, Dental Materials Research Center, Department of Operative Dentistry, Isfahan University of Medical Sciences, Isfahan, Iran

## Abstract

**Background:**

Secondary caries, degradation of the dentin-resin interface and fracture of the restoration are the most common forms of failure. The aim of this study was to assess the interfacial fracture toughness of three different adhesive approaches and to compare their fracture toughness after surface treatment with antioxidant.

**Material and Methods:**

Seventy two dentin blocks with 3×4mm dimension and 3mm thickness were prepared and attached to precured 3×4×12mm composite blocks from both sides. Six adhesive groups (N=12), All-bond universal, Scotchbond universal and Clearfil SE bond with or without antioxidant treatment (Sodium Ascorbate 10% )were applied to dentin surfaces, a 160µ polyethylene paper formed the chevron in adhesive-dentin interfaces. Chevron-notched beam fracture toughness was measured following a modified ISO 24370 standard. The uniform composite-dentin-composite block was subjected to a 4-point test in universal machine. Data were analyzed by Kruskal-Wallis, Wilcoxon signed-rank and Mann-Whitney tests (α =0.05).

**Results:**

Different adhesive approaches yielded different significant in fracture toughness rates. A significant increase of fracture toughness was observed between adhesive groups after antioxidant surface treatment. The difference in fracture toughness between Scotchbond universal and All-bond universal were significant.

**Conclusions:**

The highest value of fracture toughness was reported for Clearfil SE bond and the lowest value was found for All-bond universal. Sodium ascorbate as antioxidant surface treatment had a significant effect in increasing the fracture toughness.

** Key words:**Chevron-notched beam fracture toughness, fracture toughness, dentin, adhesive, antioxidant.

## Introduction

Secondary caries, degradation of the dentin‒resin interface and fracture of the restoration are the most common failure forms of composite resin restorations (1). As these failures appear to be largely related to the bond integrity between the adhesive and the tooth structure, or result from poor immediate adhesion, the interface strength has emerged as a key criterion for restoration performance (2).

The adhesive systems available on the market can be classified into two main categories: etch-and-rinse and those applied using self-etch strategies, and the new group of adhesives are called ‘‘Universal’’, ‘‘Multi-purpose’’ or ‘‘Multi-mode’’ adhesives (3). Single-bottle universal adhesives have been developed to formulate an optimized mix of compatible, hydrophobic, adhesive functional and hydrophilic monomers. These adhesives are polymerized form a durable and hopefully hydrophobic, bonded interface. Each monomer in universal adhesives has its own particular functions. The hydrophilic ends of monomers interact with the tooth tissues, while the hydrophobic ends interact with methacrylate-based restorative materials or cross-link with other functional and structural monomers. The hydrophilic terminal ends of the ionic phosphate group in 10-MDP turn into more hydrophobic ends while reacting with tooth tissues, and polymerized (4) 10-MDP is bonded to calcium in hydroxyapatite (Ca10(PO4)6(OH)2) by ionic bonding (5). Stable MDP-calcium salts are formed through this reaction and deposited in self-assembled nano-layers of changeable degrees and quality on the adhesive system. Molecular interaction and self-organization, coupled with the relatively hydrophobic nature of polymerized 10-MDP, make this monomer so effectual in creating adhesive interfaces that are resistant to biodegradation.In some universal adhesives, phosphate esters (R-O-PO3H2) are primary adhesive functional monomers. These monomers have the potential to bond to zirconia, metals and tooth tissues by creating non-soluble Ca++ salts (4).

The durability of the bond between dentin and the adhesive system depends largely on the structural integrity and mechanical properties of acid-demineralized collagen fibers (6). Covalent inter- and intra-molecular cross-links are the basis for stability, tensile strength and viscoelasticity of collagen fibrils (7).

The mechanical properties of collagen and its resistance to enzymatic degradation can be improved by an increase in the formation of intra- and inter-molecular and inter-microfibrillar cross-links. This can be achieved by application of various collagen cross-linkers, both synthetic and natural, on the dentin substrate prior to the bonding procedure (8,9). Naturally occurring collagen cross-linkers such as sodium ascorbate and proanthocyanidin have been reported to increase the collagen cross-linking in the sound and caries-affected dentin (9,10). The positive effect of these collagen cross-linkers on the bond strength of a self-etch adhesive to deep dentin has been reported in the literature (7). Also, it seems that the efficacy of antioxidant depends on the type of adhesive system (11).

A new method to evaluate the bond strength is fracture toughness test (12). Fracture toughness test is considered a more valid method to assess bonding effectiveness compared with conventional bond strength testing (13). However, to date, only few studies have measured fracture toughness using various methodologies (12-15).

Therefore, the purpose of the present study was to assess the fracture toughness of adhesives bonded to dentin using the modified fracture toughness ISO 24370 standard for ceramics, compared to different adhesive approaches, and to assess the effect of surface treatment on fracture toughness values.

The hypotheses tested were: 1) There is no difference in interfacial fracture toughness between different adhesive approaches and 2) Surface treatment with antioxidants has no effect on the bond strength of adhesive to dentin.

## Material and Methods

This study was approved by the Ethics Committee for Research at Isfahan University of Medical Sciences (IRB No. 393567).

The fracture toughness of adhesive–dentin interfaces was determined using a chevron-notched beam (CNB) test, adapted from the modified ISO 24370 standard to measure the fracture toughnessof ceramics.

1. Specimen preparation

Non-carious human third molars (gathered following informed consent approved by the Commission for Medical Ethics of Isfahan University of Medical Sciences) were stored in 0.2% thymol solution at 4°C and were used within 3 months after extraction. The teeth were stored in distilled water at 37°C for approximately 24 hours before dentin block preparation.

**Figure 1:**
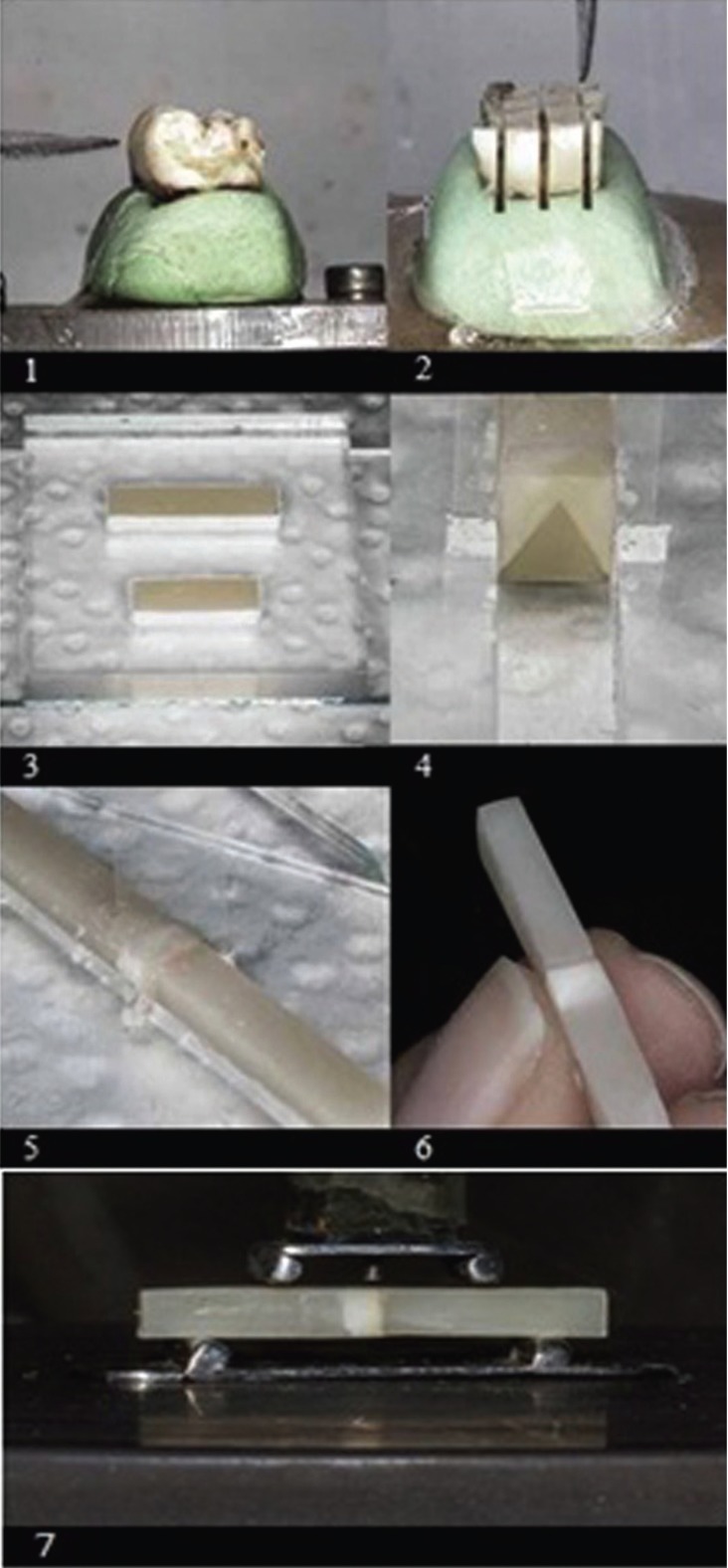
Specimen preparation: 1.cutting the third of the crown 2.preparation of dentin blocks 3×4×3 3.precured composite blocks 4.attached dentin to composite 5.fixing the chevron with polyetheln barrier 6.the uniform 3-part composite-dentin-composite. 7. 4-point test loading.

To expose a flat mid-coronal dentin surface, the occlusal third of the crown was removed by a diamond saw (CNC cutting section machine, Mashhad, Iran) using low speed and under water cooling (Fig. 1). Two rectangular dentin blocks, one for the adhesive group and the other for the antioxidant group, were cut from the tooth, measuring 3×4 mm in dimension and 3 mm in thickness. The specimens were evaluated by digital calipers. The dentin surfaces were carefully examined under a stereomicroscope (Trinocular Zoom Stereomicroscope) for the absence of enamel or pulp tissue remnants.

Then, 160-grit Opti disks (Kerr, USA) were used to produce a standardized bur-cut smear layer by grinding a thin layer of the surface. Next, the flat dentin blocks were stored in distilled water.

Two 3×4×15-mm and 3×4×12-mm rectangular composite resin (Grandio, Voco) blocks were light-cured for 30 sec with high power (≥1000 mW⁄cm2, Demi, Kerr, USA) in plexi molds prepared using a CNC laser machine and were stored in distilled water for 24 hours to help release internal stresses (Fig. 1).

The prepared 15-mm blocks consisted of 3 mm of dentin and 12 mm of composite resin. The 3×4×3-mm dentin blocks were bonded to precured 3×4×12-mm composite resin blocks with Clearfil SE Bond adhesive and flowable composite resin (Grandio, Voco) with mechanical undercuts in 3×4×15-mm plexi mold and were light-cured as a uniform block (Demi) (Fig. 1).

2. Adhesive application

The dentin blocks in group 1, bonded to the composite resin block, underwent a bonding procedure in three subgroups: A. Clearfil SE bond; B. Scotchbond Universal; and C. All-bond Universal. The adhesives were applied to dentin surfaces according to the manufacturers’ instructions ( Table 1).

**Table 1:**
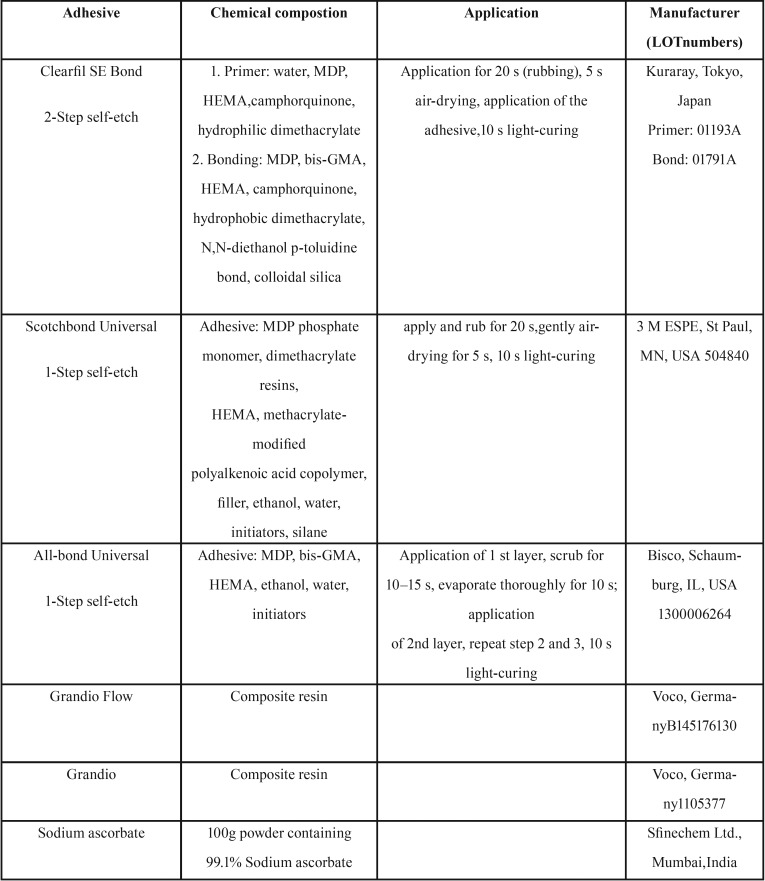
Materials tested.

3. Chevron preparation

A 160-µm polyethylene barrier was prepared by laser, with a triangular void shape located in the central cleft of a 3×4×30-mm mold. To ensure a close contact of polyethylene barrier with the adhesive before light-curing, the tip of an explorer was used to hold the barrier in close contact with the dentin surface; the adhesive was then cured for 30 sec (Demi, USA) (Fig. 1).

The pre-cured composite resin block, measuring 3×4×15 mm in dimension, was placed in the other side of mold, and this block was attached to the adhesive surface with a flowable composite; while the polyethylene chevron paper was placed between the adhesive surface and the flowable composite, and composite resin flashes were carefully controlled. The uniform three-part specimen (composite‒dentin‒composite) was carefully removed from the split mold. Then, the polyethylene barrier was removed carefully so as not to exert any stress on the adhesive‒dentin interface.

4. Antioxidant surface treatment

The dentin blocks in group 2 underwent surface antioxidant treatment with 10% sodium ascorbate for 3 min before the adhesive was applied. This was followed by the same procedures as those in group 1 in three subgroups: A1. Clearfil SE bond; B1.Scotchbond Universal; and C1. All-bond Universal.

5. Fracture toughness test

Immediately after preparation of the chevron notch, the specimens were ready for loading into a universal testing machine (Electromechanical Testing Machine, Switzerland). The specimens were tested under a 4-point bend test setup with a crosshead speed of 0.05 mm/min. The outer and inner spans were 20 and 10 mm, respectively (Fig. 1). The exact dimensions of the chevron notch were similar in all the specimens, from which the minimum stress intensity factor coefficient (Ymin) was calculated for each specimen individually according to the ISO standard. Using this Ymin, the interfacial fracture resistance was calculated in MPa m1/2. All the fractured surfaces were processed for scanning electron microscopy evaluation (Leo Electron Microscope, LTD, Cambridge, England) using common preparation procedures, including fixation, dehydration and gold-sputter coating, to determine fracture location, crack propagation and possible imperfection.

6. Study setup and statistical analysis

The interfacial fracture toughness of the three adhesives was measured ( Table 2). For Scotchbond Universal, the adhesive was used only in the self-etch mode. For the CNB fracture toughness test, 12 teeth were used for each adhesive, each yielding 2 sticks: one for the adhesive and the other for the surface antioxidant treatment.

**Table 2:**
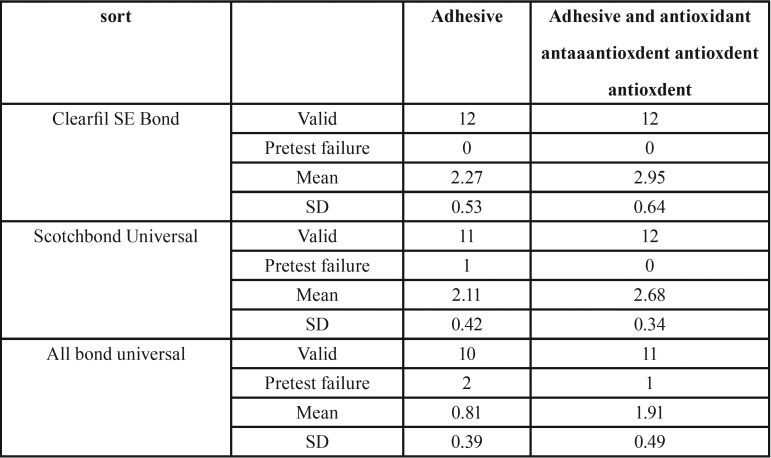
CNB fracture toughness results ( MPa m1/2).

The interfacial fracture toughness data were analyzed by Kruskal-Wallis test to compare different adhesive approaches. Wilcoxon signed-rank test was used to evaluate the effect of antioxidant treatment on the fracture toughness values. Mann-Whitney test was used for pair-wise comparisons of different adhesive approaches after antioxidant treatment.

## Results

The overall values of all tested properties were shown in  Table 2. Kruskal-Wallis test showed the different adhesive approaches have significant different of fracture toughness value.

Wilcoxon signed-rank test was applied to compare the antioxidant effect on fracture toughness for each adhesive. The significant increase of fracture toughness was shown for all adhesive groups with antioxidant surface treatment ( Table 3).

**Table 3:**
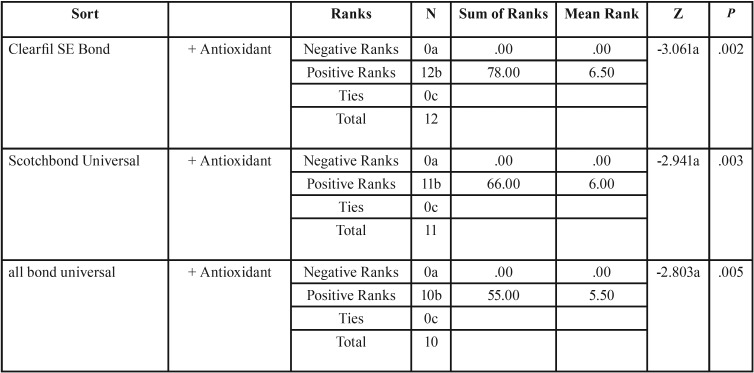
The results of Wilcoxon signed ranks test to compare the effect of adhesive with or without antioxidant on fracture toughness in different adhesive approaches.

To evaluate the difference induced by antioxidant treatment in fracture toughness for all adhesives,Kruskal-Wallis test was run, which showed a significant difference (*p*=0.026) in fracture toughness among three groups after antioxidant treatment.

Mann-Whitney test for pair-wise comparison of adhesives after antioxidant treatment showed a significant difference in fracture toughness between Scotchbond Universal and All-bond Universal (*p*=0.006). The comparison between Clearfil SE bond and other groups indicated no significant difference. To fix the overall rate of α, we used Bonferroni adjustment and considered α=0.05/3 .

**Figure 2:**
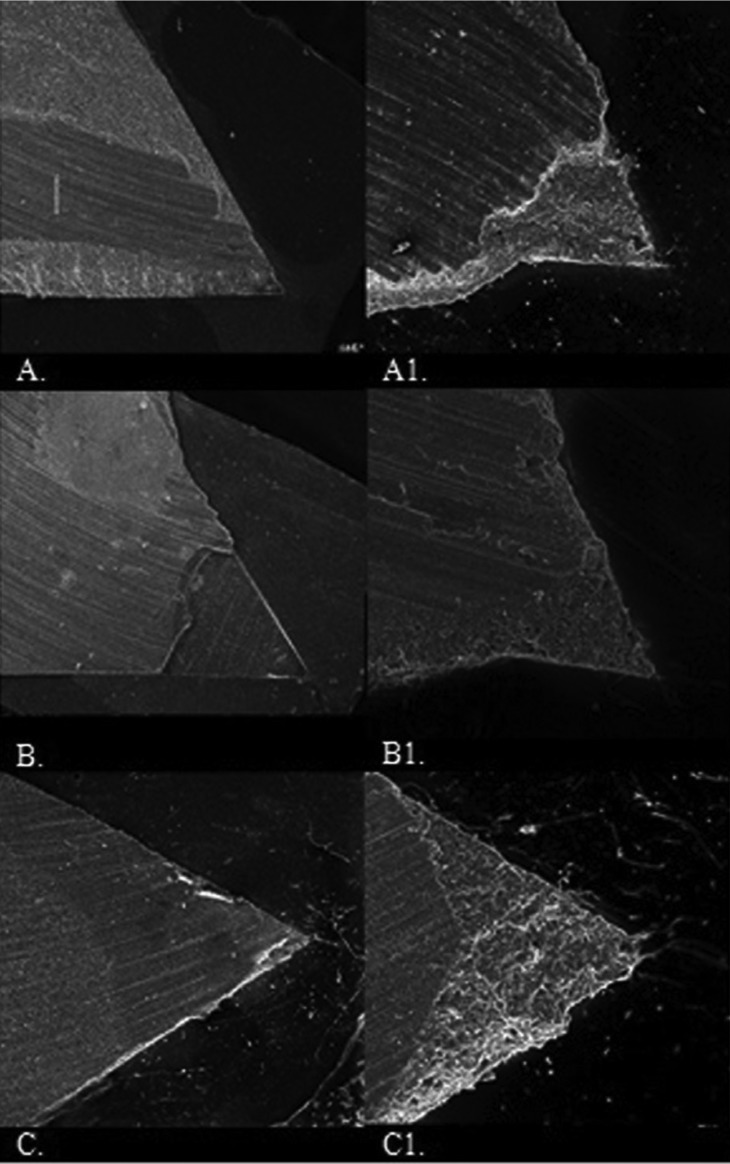
A. Overview of the fractured surface of a Clearfil SE Bond specimen. At the chevron tip, the specimen failed very close to the resin–dentin interface, while further away, fracture deviated into the adhesive resin and composite. A1. After antioxidant surface treatment at the chevron tip, failure occurred at resin dentin interface and the crack deviated toward the resin composite. B. Overview of the fractured surface of a ScotchbondUniversal specimen. At the chevron tip, the specimen fractured at the adhesive–dentin interface and the crack deviated toward the adhesive resin and composite at the end of the fracture surface. B1.With antioxidant treatment, the pattern of fracture changed and failure occurred at the resin–dentin interface. Although the flaw (tip of the chevron notch) was positioned at the interface, the crack deviated toward the resin composite. C. Overview of a fractured surface of an All-bond Universal specimen. The entire surface failed at the resin–dentin interface. C1. After antioxidant treatment, the initiation of crack was at the chevron tip located in the hybrid layer. Higher magnification of the chevron tip area showed the specimen failed at the interface exposing the hybrid layer, and some resin tags were observed at the tip of chevron, but it propagated almost completely along the resin–dentin interface.

The SEM overview of the fractured surface of specimens is depictedin Figure 2.

## Discussion

Both hypotheses in this study, i.e. (1) there is no difference in interfacial fracture toughness between different adhesive approaches and (2) surface treatment with anti-oxidant has no effect on bond strength of adhesive to dentin, were refuted.

The ISO 24370 standard is intended to determine the fracture toughness of monolithic ceramic blocks and therefore could not be directly applied to adhesive–dentin interfaces. However, in dental materials research, most fracture toughness tests are based on a chevron notch approach (14). Most methods require the use of molds and/or a tape to prepare such a notch at the interface (12). Another advantage of the present CNB interfacial fracture toughness set-up was that the adhesive was applied to dentin blocks measuring 3 mm in thickness, in contrast to, for instance, an 0.85-mm-thin dentin disc (16) that is very prone to severe dehydration. Any cutting procedure for the design of chevron might induce stresses in the specimen. If the methods of chevron preparation in such studies are implemented with care, more reliable results will be achieved.Analysis of the CNB interfacial fracture toughness of specimens suggests good loading situation; at many areas, the specimen failed at the adhesive-dentin interface and nearly linear crack fronts were seen (17).

In addition, according to the data in this study, which are a little different from the data reported in Demunck’s study (12), the high mean fracture toughness in this study might be attributed to the following reasons:

The samples were loaded only a short time after they were prepared.

Cutting methods were not used to prepare the specimen. It is noteworthy that the shearing and slipping process of the saws in the cutting method decreases the fracture toughness itself by producing stresses at dentin‒adhesive interface.

The analysis of failed specimens in fracture toughness test reveals that the origin of failure is always located at the adhesive–dentin interface because a flaw, the chevron notch, is placed exactly at this interface. This is a key feature as these materials behave in a brittle manner, so purposely placing a flaw in a controlled position and direction relative to the applied load enables the measurement of interfacial fracture (12).

The self-etch adhesives used in this study, with pH values around 2, like Scotchbond Universal (3M) and Clearfil SE Bond (CSE; Kuraray, Osaka, Japan) partially demineralized the dentin, leaving a substantial amount of hydroxyapatite crystals around the collagen fibrils (18), and performed well in fracture toughness test with mean values of 2.27 and 2.11 MPa m1/2, respectively. But the CNB interfacial fracture toughness of the one-step self-etch adhesive All-bond Universal exhibited significantly lower fracture toughness values than Clearfil SE Bond and Scotchbond Universal at the characteristic mean strength of 0.81 MPa m1/2. This is probably related to the relatively shallow interaction with the underlying dentin.

**Figure 3:**
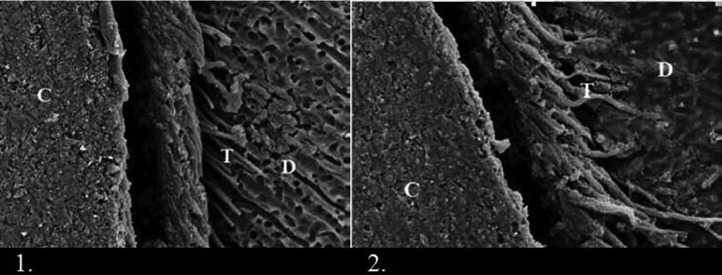
All-bond Universal before and after antioxidant treatment: 1: see the shallow interaction of ultra-mild adhesive with dentin surface. 2: after antioxidant treatment, more resin tags were formed and the zone of interaction became wider.

The main difference between this adhesive and others is the higher pH (>3, technical information from Bisco) and a higher content of hydrophobic monomers in All-bond Universal adhesive. Such an ultra-mild etching capacity, as observed in our SEM images too (Fig. 3), may suffer from smear-layer interference and thus explain the lower fracture toughness. In another study, the lowest interfacial fracture toughness onto enamel was found for the adhesives applied following a one-step self-etch approach (15).

As these materials, i.e. Scotchbond Universal (3M), Clearfil SE Bond (CSE; Kuraray, Osaka, Japan) and All-bond Universal, have 10-methacryloyloxydecyl dihydrogen phosphate monomer (MDP) in their composition, they bond to dentin chemically (19). Yoshida et al showed that an effective chemical interaction occurs between MDP and hydroxyapatite, forming a stable nano-layer that could form a stronger phase at the adhesive interface, thereby increasing the mechanical strength of the adhesive interface.In addition, stable MDP-Ca salt deposition along with nano-layering may explain the high bond stability, (20) which has previously been proven (19,21).

Chemical bonding between 10-MDP and dental hard tissues may play an important role in providing stable and durable interfaces. The chemical bonding provided by the 10-MDP molecule in the primer, together with the excellent mechanical properties and high conversion rate of its filled hydrophobic resin, resulted in a very good clinical behavior of Clearfil SE Bond (CSE; Kuraray, Osaka, Japan) during an 8-year period (19). In this study, Clearfil SE Bond showed the best performance in fracture toughness test,with amean of 2.27MPa m1/2.

Although Scotchbond Universal contains less 10-MDP than Clearfil SE Bond, it contains a polyalkenoic acid copolymer. This copolymer was first used in the composition of Vitrebond (3M ESPE), also known as Vitrebond copolymer or VCP. This copolymer bonds chemically to the calcium in hydroxyapatite (22). For self-etch adhesives, chemical bonding between polycarboxylic monomers (such as VCP) and hydroxyapatite plays a crucial role in their bonding mechanism. Over 50% of the carboxyl groups in the polyalkenoic acid copolymer are capable of bonding to hydroxyapatite. Carboxylic groups replace phosphate ions in the substrate and make ionic bonds with calcium (23). This is areason for thegood result offracture toughness test using Scotchbond Universal,with 2.11 MPa m1/2.

 With these two chemical bonding mechanisms in mind, the clinical behavior of Clearfil SE Bond and Scotchbond Universal in our study may have been the result of: 1) the chemical bonding ability of both 10-MDP monomer (5,24) and VCP to hydroxyapatite; 2) the protective effect of calcium-MDP (Ca-MDP) salt (19), as the Ca-MDP salt is one of the most hydrolytically stable salts (5); and 3) the formation of a submicron micromechanical interlocking at the dentin surface by SU (20). The monomer 10-MDP is adsorbed onto hydroxyapatite in a regularly layered structure at the hydroxyapatite surface (nano-interaction) (20,24), and at the same time decalcifies hydroxyapatite (5).

Two main methods for increasing the dentin/resin interface properties have to be considered: the continuing improvement/development of new adhesive systems and the establishment of tissue engineering/biomimetic approaches to improve the intrinsic properties of the substrate. Intrinsic collagen cross-links provide the tensile properties of collagen molecules. The use of extrinsic collagen cross-linking agents can induce additional formation of inter- and intra-molecular cross-links (25). Selective cross-linking agents have been demonstrated to increase the ultimate tensile strength and elastic modulus of demineralized dentin (26).

Reactive oxygen species can ‘abstract’ a hydrogen from ascorbate, which becomes monodehydroascorbate and soon gains another electron to become dehydroascorbate (27). Sodium ascorbate suppresses the denaturing effect of etching on dentin collagen (28), offering protection against the degradation of composite–dentin bond. In addition, sodium ascorbate is an important component in the synthesis of hydroxyproline and hydroxylysine in collagen. Hydroxyproline serves to stabilize the collagen triple helix, and hydroxylysine is necessary for the formation of intermolecular cross-links in collagen (29). Antioxidant-doped adhesives have positive effects on adhesive interface durability (30). In this study, surface treatment with sodium ascorbate significantly increased the fracture toughness values of all the adhesives.It has been known that ascorbic ions form relatively stable complexes with calcium ions (31). In aqueous solutions, ascorbic acid and its salt undergo ionization to form singly- and doubly-charged anions, and each of these anionic species may couple with calcium ions to form a complex of 1:1 type. Such a property can help reduce the availability of ionic calcium in the presence of a relatively large quantity of ascorbate. This reducing agent interfered with dentin hybridization when ClearFil SE bond and Scotch bond wereused as ascorbate ions,which might have scavenged calcium ions along the dentin surface (27). This binding depletion at the bonded interface served as stress raisers that led to a minor decrease in fracture toughness in their absence. However, TheAll-bond Universal adhesive contains less 10-MDP and increases collagen cross-links with sodium ascorbate.Thefracture toughness value is higher in All-bond Universaladhesive in comparison withother bondings in this study. Also, the increased fracture toughness in All-bond Universal adhesive is clearly observed with antioxidant treatment. The result of this bonding may be due toultra-mild pH=3.2, which has very little interference with the smear layer. Treatment with antioxidant will increase the possibility of more interference with the smear layer, resulting in the formation of more resin tags, as evidenced by SEM results (Fig. 3).

In general, the increase in fracture toughness value by sodium ascorbate treatment is the result of equilibrium between:

1. the increased stability and cross-linking of collagen network that improved the quality of the hybrid layer

2. the quantity of calcium ions available in the hybrid layer for chemical interaction with 10-MDP or other active molecules.

## Conclusions

Under the limitations of the current *in vitro* study, it can be concluded that Scotchbond Universal, as a new adhesive agent, exhibitsa good performance in the CNB fracture toughness test in comparison with Clearfil SE Bond as the gold standard of self-etch adhesives. Sodium ascorbate as an antioxidant for surface treatment significantly increases the fracture toughness values and hasa positive effect on the hybrid layer quality.
